# The *MYH7* c.2770G > A (p.Glu924Lys) mutation exhibits phenotypic heterogeneity in hypertrophic cardiomyopathy (HCM) and restrictive cardiomyopathy (RCM): a case report

**DOI:** 10.1186/s12872-025-04943-x

**Published:** 2025-07-16

**Authors:** Yuanyuan Han, Haiyan Wang, Hongsheng Zhang, Manman Wang, Lijun Gan, Fanhua Meng

**Affiliations:** 1 Department of Cardiology, Affiliated Hospital of Jining Medical University, Shandong, China; 2 Shandong Provincial Key Medical and Health Discipline of Cardiology Affiliated Hospital, Jining Medical University, Shandong, China; 3 Key Laboratory of Cell and Biomedical Technology of Shandong Province, Shandong, China

**Keywords:** *MYH7*, Gene mutation, Hypertrophic cardiomyopathy, Restrictive cardiomyopathy, Case report

## Abstract

This study reports on a Chinese Han cardiomyopathy family line carrying the *MYH7* c.2770G > A (p.Glu924Lys) mutation. This mutation has been shown to result in cross-generational phenotypic heterogeneity between hypertrophic cardiomyopathy (HCM) and restrictive cardiomyopathy (RCM). The proband was a 17-year-old male diagnosed with hypertrophic obstructive cardiomyopathy (HOCM) due to post-exercise syncope, which resolved after septal septectomy. Family lineage analysis revealed that the mother (RCM, NYHA class III) and uncle (RCM, NYHA class IV) of the proband presented with bi-atrial enlargement and reduced systolic function (LVEF 45%), whereas aunts and cousins (*n* = 3) had asymptomatic hypertrophic non-obstructive cardiomyopathy (HNCM). Whole exome sequencing identified six family members carrying the *MYH7* c.2770G > A heterozygous mutation with an ACMG rating of probable pathogenic (PM2 + PP3 + PP5). The subjects carrying the mutation exhibited three distinct phenotypes: HOCM (1 case), HNCM (3 cases), and RCM (2 cases). However, the mutation locus did not fully co-segregate with the observed phenotypes. The study hypothesises that the *MYH7* c.2770G > A mutation can lead to transgenerational and multisubtype cardiomyopathy phenotypic heterogeneity, and that the mechanism may be related to transcriptional regulation and epigenetic modifications. It is imperative that early genetic screening and clinical intervention are implemented to enhance the prognosis of affected families.

**Supplementary Information:**

The online version contains supplementary material available at 10.1186/s12872-025-04943-x.

## Introduction

Cardiomyopathy is a heterogeneous group of diseases characterized by structural and functional abnormalities of the myocardium. Its diagnosis requires the exclusion of secondary factors such as coronary artery disease, hypertension, valvular disease, and congenital heart disease, and its clinical manifestations include heart failure, malignant arrhythmia, and sudden cardiac death [1]. Based on morphofunctional phenotype, cardiomyopathies are mainly classified into hypertrophic cardiomyopathy (HCM), dilated cardiomyopathy (DCM), non-dilated LV cardiomyopathy (NDLVC), arrhythmogenic right ventricular cardiomyopathy (ARVC), and restrictive cardiomyopathy (RVC) [1, 2].Research has demonstrated that approximately 40% of HCM and 30% of RCM can be attributed to mutations in well-defined causative genes [1, 2]. The MYH7 gene, which encodes the β-myosin heavy chain, is the primary causative gene for familial HCM, accounting for approximately 33% of familial HCM cases. It is also associated with approximately 5% of DCM cases and is linked to RCM [3]. Additionally, the MYBPC3 gene, which encodes cardiac myosin-binding protein C, is the second most common causative gene for HCM. Truncation mutations in this gene can lead to sarcomere contraction dysfunction [4]. In the pathogenic mechanism of RCM, mutations in the TNNI3 (encoding cardiac troponin I) and TNNT2 (encoding cardiac troponin T) genes affect troponin complex function and participate in regulating myocardial diastolic dysfunction [4, 5]. Abnormal filament protein aggregation caused by FLNC gene mutations, protein quality control defects caused by BAG3 gene mutations, and CRYAB gene mutations causing molecular chaperone dysfunction have all been shown to be associated with protein pathological changes in RCM [4, 5]. These gene mutations interfere with sarcomere structure, calcium homeostasis regulation, and protein degradation pathways, collectively forming the complex genetic pathogenic spectrum of HCM/RCM.

The *MYH7* gene is located on the long arm of chromosome 14 (14q11.2) and encodes a β-myosin heavy chain that is a key component of cardiomyocyte contractile function. The gene's mutations are predominantly concentrated in the head function domain (exons 3–23), and missense mutations in different regions can lead to abnormal myofibroblast mechanics by interfering with actin binding or ATPase activity [6]. It has been demonstrated that mutations in the head-rod binding region are predominantly associated with early-onset severe HCM, whereas rod-tail mutations may result in phenotypic heterogeneity [7]. Despite the strides made in *MYH7* genotype–phenotype association studies, the mechanism by which a single mutation can manifest as both HCM and RCM within a family line remains to be elucidated [8]. This study utilized clinical phenotyping and whole exome sequencing of a multigenerational cardiomyopathy family line, revealing that the c.2770G > A (p.Glu924Lys) mutation in the *MYH7* gene was significantly associated with HCM and RCM phenotypic heterogeneity.

## Case description

### Clinical characteristics of pre-existing witnesses

**Fig. 1:**
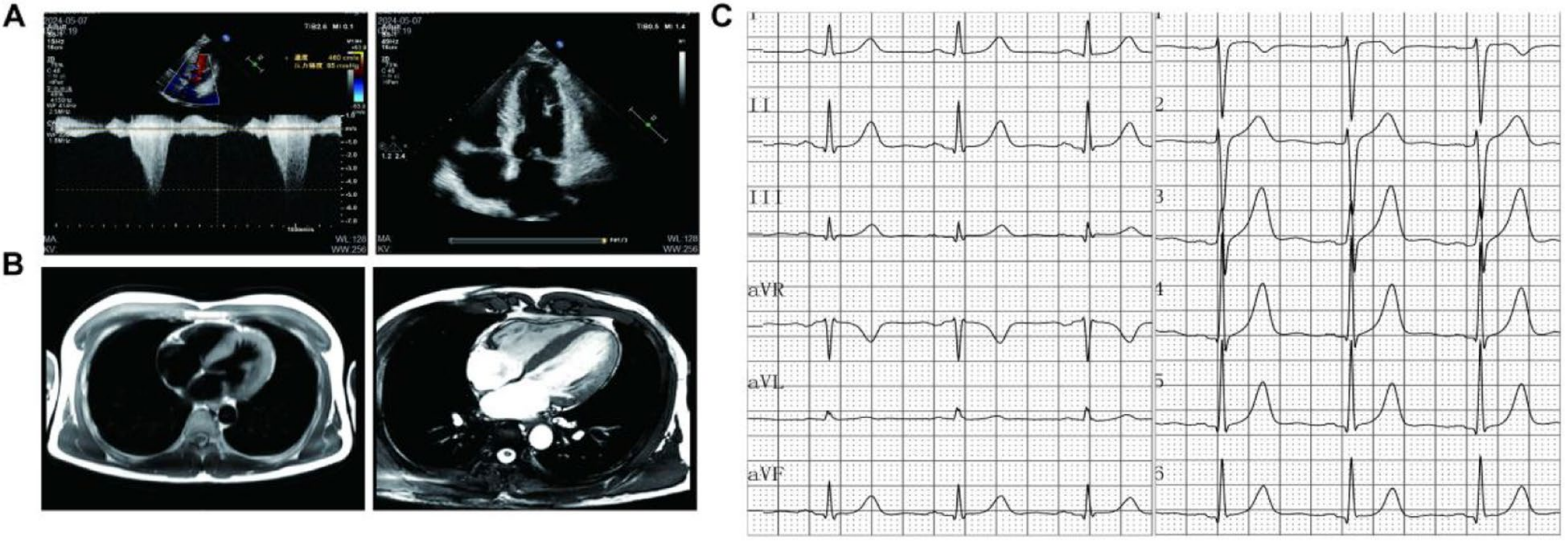
Cardiac colour Doppler and electrocardiogram in preexisting patients. **A**, cardiac colour Doppler revealed significant asymmetric hypertrophy of the basal segment of the interventricular septum, narrowing of the left ventricular outflow tract, and anterior motion of the anterior mitral leaflet during systole (positive SAM sign). **B** Cardiac magnetic resonance: The presence of inhomogeneous thickening of the left ventricular wall (predominantly in the septal septum), narrowing of the left ventricular outflow tract, and diffusely elevated T1 values of the left ventricular myocardium were observed. **C**, Electrocardiogram results suggestive of sinus arrhythmia were noted

The Pre-existing witness (III-2) was a 17-year-old male who was previously fit and had no family history of cardiovascular disease. Recurrent post-exercise blacking-out episodes had not been accorded sufficient seriousness in the preceding two years. On this occasion, the patient was admitted to the hospital as an emergency case following a sudden loss of consciousness that lasted approximately 30 s whilst participating in basketball training. On examination, the patient's blood pressure was recorded at 115/82 mmHg, the heart rate was 98 beats per minute and was found to be rhythmic, and a grade 3/6 systolic jet-like murmur was audible in the 3–4 intercostal space at the left sternal margin, which increased to grade 4/6 after the Valsalva maneuver. Transthoracic echocardiography revealed significant asymmetric hypertrophy of the basal segment of the interventricular septum (maximal thickness 18 mm, normal reference value ≤ 12 mm), a peak left ventricular outflow tract (LVOT) pressure difference of 82 mmHg (at rest), anterior mitral leaflet anterior motion during systole (positive SAM sign), and a left ventricular ejection fraction (LVEF) of 64% (Fig. 1A). Cardiac magnetic resonance (CMR) delayed enhancement imaging revealed elevated T1 values in the left ventricular myocardium (1,244–1,419 ms, normal < 1,100 ms) (Fig. 1B). Three episodes of non-sustained ventricular tachycardia (NSVT, lasting for up to five beats) were documented on a 24-h ambulatory electrocardiogram. According to the 2022 ESC diagnostic criteria for hypertrophic cardiomyopathy [1], the diagnosis of hypertrophic obstructive cardiomyopathy (HOCM) was confirmed, and transaortic septal cardiomyotomy (ASA) was performed. Postoperative repeat TTE showed a decrease in LVOT pressure difference to 12 mmHg and complete symptomatic relief.

### Phenotypic distribution and clinical characteristics of family members

**Fig. 2:**
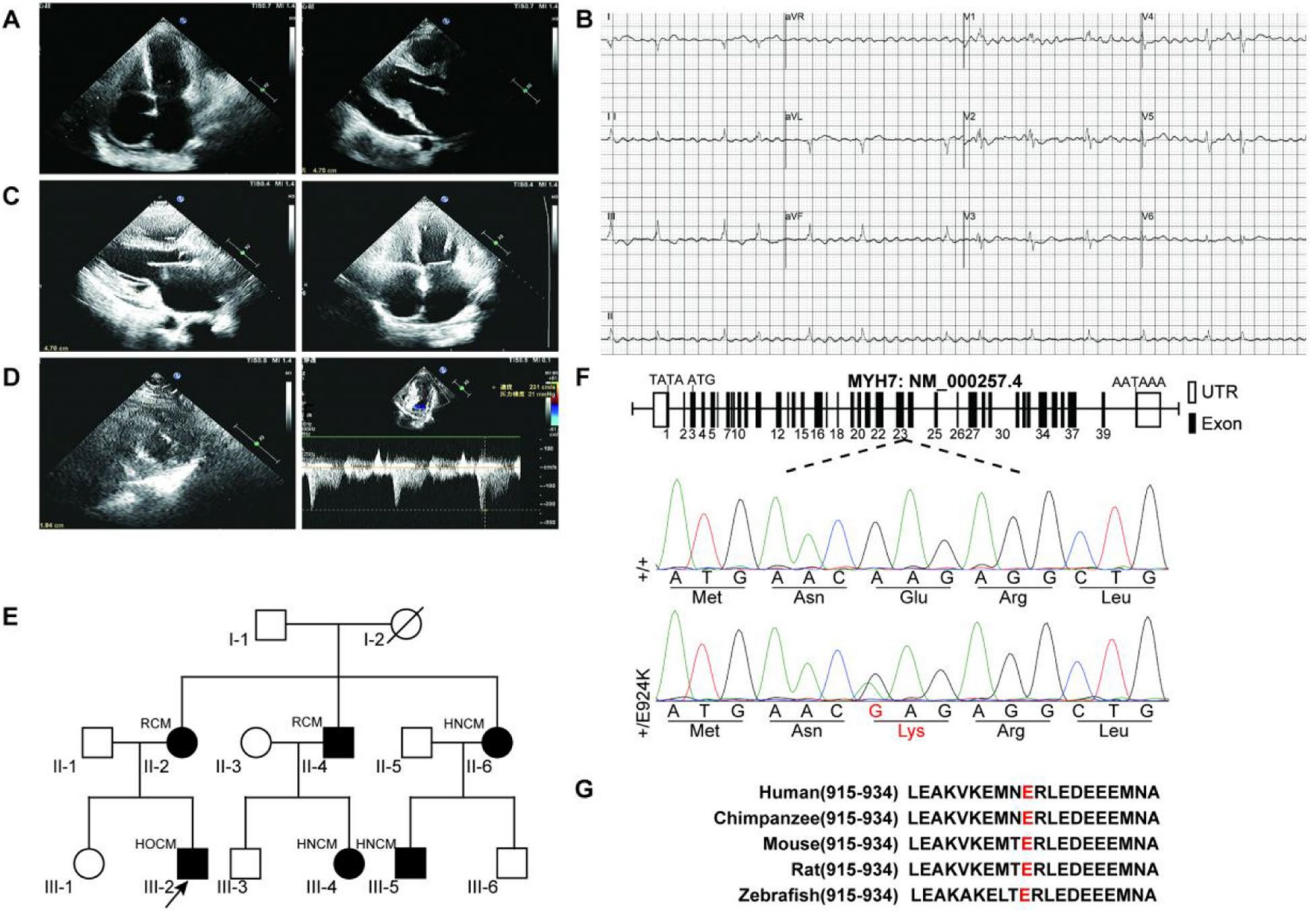
Phenotypic distribution and clinical characteristics of family members. A**-**B, Proband’s mother (II-2), ECG: atrial fibrillation. Echocardiography: biatrial enlargement, normal ventricles, uncoordinated LV movement, reduced LVEF (45%), pericardial effusion. **C**, Uncle (II-4), ECG: atrial fibrillation. Echocardiography: biatrial enlargement, coventricular septum motion, reduced LVEF (44%), pericardial effusion. **D**, Asymptomatic cousin (III-3): echocardiography shows hypertrophic non-obstructive cardiomyopathy with left atrial enlargement, thickened LV apex/septum/anterior wall, ground-glass echo enhancement, disordered myocardial texture, and reduced contractility. **E**,1 Analysis of a Chinese Han family with cardiomyopathy. The family carries the E924K heterozygous mutation (+/E924K). Squares and circles represent males and females, respectively. Roman numerals represent generations, and Arabic numerals represent specific family members. **F**, Genomic structure of the human *MYH7* gene. The G to A substitution at base 2770 of exon 23 leads to the E924K mutation.**G,** Alignment of sequences flanking the E924 residue (indicated by red) of *MYH7* in various species

A total of 14 family members were included in this study for clinical and genetic screening, of whom 6 carried the *MYH7* c.2770G > A mutation. The clinical features of each carrier are outlined below: II-2 (the 46-year-old mother of the first witness) presented with progressive exertional dyspnea (NYHA class III) with nocturnal paroxysmal dyspnea. Transthoracic echocardiography (TTE) revealed significant bi-atrial enlargement (left atrial diameter 58 mm, right atrial diameter 49 mm), normal left ventricular end-diastolic internal diameter (45 mm), interventricular septal thickness of 11 mm, LVEF 45%, and no LVOT obstruction, which was consistent with the criteria for the diagnosis of RCM [1]. II-4 (the patient's uncle, aged 38 years) presented with palpitations and chest tightness. ECG showed atrial fibrillation (ventricular rate 110 beats/min). TTE showed bi-atrial enlargement (left atrium 48 mm, right atrium 47 mm) and LVEF 44%. Right heart catheterization showed left ventricular end-diastolic pressure (LVEDP) of 22 mmHg (normal < 15 mmHg), confirming the diagnosis of RCM (NYHA class IV). II-5 (aunt, aged 51 years), III-3 (cousin, aged 15 years), and III-5 (cousin, aged 19 years) were found to be asymptomatic. TTE revealed asymmetric hypertrophy of the left ventricular wall (septal thickness 12–15 mm). LVOT peak pressure difference was found to be less than 30 mmHg, and a diagnosis of hypertrophic non-obstructive cardiomyopathy (HNCM) was made (see Fig. 2A-D). It is noteworthy that all carriers were found to be free of secondary factors such as hypertension and diabetes.

### Gene detection and functional verification

Whole-exome sequencing (WES) was performed on the proband and their family members, with a focus on known pathogenic genes associated with cardiomyopathy. WES detected a heterozygous mutation c.2770G > A (p.Glu924Lys) in the *MYH7* gene in the preterm, which was verified to be segregating within the family by Sanger sequencing (Fig. 2E-2F). Bioinformatics analysis revealed that the p.Glu924 locus is highly conserved in mammals, suggesting its functional importance (Fig. 2G). PolyPhen-2 predicted the mutation to be'probably pathogenic'(score 0.998) with a REVEL score of 0.89 (threshold > 0.75), which is consistent with the American College of Medical Genetics and Genomics (ACMG) probable pathogenicity rating (PM2 + PP3 + PP5) [9]. The subsequent phenotype-genotype association analysis demonstrated a 2:1 ratio of HCM to RCM in mutation carriers (4 cases of HCM, including 1 case of obstructive HCM and 3 cases of non-obstructive HCM; 2 cases of RCM). The age of onset exhibited a bimodal distribution, with a mean of 42 ± 5.6 years in the RCM group and 21 ± 7.3 years in the HCM group (*p* = 0.11) (Table 1).

**Table 1:** General information on cardiomyopathy families

Patient no	Mutation	Age	Gender	First symptom	Disease
I-1	Not found	75	Male	None	None
I-2	unknown	74	Female	unknown	unknown
II-1	Not found	54	Male	None	None
II-2	p.E924K(c.2770G > A)	53	Female	amaurosis,syncope	RCM
II-3	Not found	48	Female	None	None
II-4	p.E924K(c.2770G > A)	47	Male	heart failure	RCM
II-5	p.E924K(c.2770G > A)	50	Female	heart failure	HCM
II-6	Not found	50	Male	None	None
III-1	Not found	21	Female	None	None
III-2	p.E924K(c.2770G > A)	17	Male	None	HCM
III-3	p.E924K(c.2770G > A)	16	Female	None	HCM
III-4	Not found	23	Male	None	None
III-5	p.E924K(c.2770G > A)	24	Male	None	HCM
III-6	Not found	18	Male	None	None

## Discussion

This study is pioneering in its identification of the *MYH7* gene mutation c.2770G > A (p.Glu924Lys) in Han Chinese families, thus providing a valuable addition to the existing body of knowledge surrounding the coexistence of obstructive HCM, non-obstructive HCM, and RCM across generations. This finding serves to further refine our comprehension of the genotype–phenotype relationship in cardiomyopathy, thereby providing novel evidence to inform genetic counseling. It is noteworthy that no mutations in genes such as *DES*, *FLNC*, and *CRYAB* were identified in the patients of this family. This is in contrast to recent findings that have confirmed these genes to be closely associated with RCM pathogenesis [5].Structural biology analyses demonstrated that the mutation is located in the β-myosin heavy chain rod tail structural domain, which plays a pivotal role in myocardial ganglion assembly and mechanical signalling. Its pathogenicity is substantiated by the ACMG classification system (PM2 + PP3 + PP5), and lineage studies corroborate that the mutation is fully ectopically expressed (100%), has an early onset (onset at 6–16 years of age), and has a high lethality (age of sudden death is concentrated in the age range 20–25 years). 25 years of age). Although the mutation was first identified at Harvard Medical School in cases of disseminated disease (with negative parental genotypes, suggesting the possibility of a de novo mutation), this family and the family studies reported from Sweden and China have shown that the mutation is fully episodic (100%), early-onset (onset at 6–16 years of age), and highly lethal (sudden death in the age group of 20–25 years) [5, 10–13].

Pathogenic variants at different loci of the *MYH7* gene can induce clinical phenotype heterogeneity through multiple molecular mechanisms, with their pathophysiological basis involving interactions between epigenetic regulatory networks, calcium signal transduction imbalance, and mutant protein dose effects. First, epigenetic modifications serve as a core regulatory layer independent of DNA sequence variations, dynamically regulating gene transcription activity and transcription factor recruitment efficiency through mechanisms such as DNA methylation pattern remodeling, histone post-translational modifications, and chromatin conformation changes. Existing evidence suggests that the phenotypic diversity of cardiomyopathy is not only attributed to coding sequence variations but is also closely associated with abnormal reorganization of the epigenetic regulatory network [15]. In this study, despite all carriers harboring the same *MYH7* c.2770G > A mutation, clinical subtypes exhibited significant heterogeneity, suggesting that epigenetic modifications may be involved in the phenotypic differentiation process. Future research urgently requires the construction of an integrated framework combining transcriptomics, multi-omics epigenetic maps, and three-dimensional genomics analysis, combined with polygenic risk scoring (PRS) and expression quantitative trait locus (eQTL) mapping, to systematically elucidate the mechanisms by which epigenetic regulatory networks contribute to phenotypic plasticity.Second, abnormal calcium signal transduction constitutes a key regulatory node in the differentiation of cardiomyopathy phenotypes. As the core signal molecule in myocardial excitation–contraction coupling, calcium ion homeostasis imbalance directly mediates the transition of contraction phenotypes by remodeling the sensitivity of myosin filaments to calcium ions [16–19]. Upregulation of calcium sensitivity can induce myocardial overcontraction, driving the progression of the HCM phenotype [16, 17]; conversely, downregulation of calcium sensitivity leads to depletion of contractile reserve, triggering the characteristic contractile dysfunction of DCM [18, 19]. Studies have shown that patients with *MYH7* mutation-associated HCM exhibit heterogeneity in calcium channel sensitivity, which may be associated with differences in mutation sites or drug responses [18]. Notably, sarcomere structural calcium channel dysfunction and abnormal cytoskeletal mechanical transmission may exacerbate the ventricular remodeling process in DCM. Although this study did not identify peripheral blood calcium metabolism abnormalities, there is an urgent need to establish a myocardial cell-specific calcium signaling detection system to deeply elucidate the molecular dialogue mechanisms between mutation sites and calcium signaling regulatory networks, providing a theoretical basis for precise treatment with calcium sensitizers or calcium antagonists.Third, there is a significant correlation between protein dosage effects and clinical phenotype patterns. Heterozygous mutations preferentially lead to HCM phenotypes through a dominant negative effect mechanism, while homozygous mutations may induce DCM phenotypes through toxic protein aggregation effects [20]. Notably, this study did not observe a direct association between mutant allele load and phenotypic classification, which may be related to limitations in family sample size and the presence of regulatory factors such as epigenetic modifications. Future studies could utilize induced pluripotent stem cell (iPSC) technology to construct disease models and analyze the dynamic effects of mutant protein dose–response on the electrophysiological characteristics and contractile mechanics of cardiomyocytes at the single-cell resolution level. Fourth, polygenic interaction networks significantly amplify the phenotypic heterogeneity of cardiomyopathy. Co-mutations in *MYH7* and *TNNT2* synergistically enhance the risk of sudden cardiac death, with mechanisms involving dysfunction of the sarcomere protein complex and abnormal calcium signal desensitization [21, 22]. Notably, *TTN* truncation variants (TTNtv), the primary genetic driver of DCM, can disrupt sarcomere mechanical integrity and cytoskeletal signaling, thereby synergistically amplifying the pathogenic effects of *MYH7* mutations [23]. Fifth, genome-wide association studies (GWAS) have identified multiple phenotypic regulatory loci, such as *SLC6A6* variants associated with DCM, which disrupt myocardial energy metabolism, and *SMARCB1* variants that destabilize chromatin structure, collectively shaping phenotypic diversity [25–26]; *SVIL* loss-of-function variants associated with HCM are closely related to reduced energy utilization efficiency in sarcomeres [23]. PRS integrate the pleiotropic genetic signals identified in GWAS and can explain 27% of phenotypic variation in DCM risk prediction, particularly demonstrating significant predictive efficacy for left ventricular remodeling phenotypes (OR = 1.53) [24]. These findings emphasize that the phenotypic heterogeneity of cardiomyopathy results from the combined effects of single-gene main effect variants, polygenic modifying effects, epigenetic regulatory networks, and environmental exposure factors.

In comparison with preceding studies, this family line exhibits distinctive clinical characteristics, encompassing considerable phenotypic heterogeneity and a broad age range of onset (16–46 years). These features serve to confirm the pleiotropic nature of *MYH7* mutations and also support the role of gene-environment interactions in the disease process. For family members carrying the *MYH7* c.2770G > A mutation, a multidimensional surveillance system is recommended, including annual echocardiograms to assess the progression of ventricular remodelling, ambulatory electrocardiograms to capture arrhythmic events, and biomarkers to track the extent of myocardial damage. Concurrently, management strategies should be developed based on genotype specificity, such as prioritizing AF prevention for RCM phenotypes and early assessment of septal ablation indications for obstructive HCM. The present study is subject to the following limitations: firstly, the small size of the family line limits statistical validity, which may result in certain associations or differences not being detected; secondly, the lack of proteomics data limits the in-depth understanding of mutations affecting the contractile function of cardiomyocytes; furthermore, the effects of environmental factors on phenotypic differentiation were not systematically assessed, and future studies should delve deeper in this area.

## Conclusion

In this study, the pathogenicity of the *MYH7* c.2770G > A (p.Glu924Lys) mutation was confirmed for the first time in a Chinese polyphenotypic cardiomyopathy family line, thereby revealing its clinical features leading to significant phenotypic heterogeneity and a propensity for rapid progression. This finding extends the *MYH7* genotype–phenotype association spectrum and provides a valuable foundation for genetic counselling and personalized treatment. Future studies can further resolve the molecular mechanisms by which this mutation affects cardiomyocyte function and assess the impact of environmental factors on phenotypic differentiation through the establishment of iPSC models and single-cell sequencing technology.

## Supplementary Information

Supplementary Material 1

## Data Availability

The article and its Supplementary Material encompass the pivotal original contributions outlined in the study. For any additional queries or inquiries, kindly reach out to the corresponding author for further clarification.

## Abbreviations

HCM: Hypertrophic cardiomyopathy
RCM: Restrictive cardiomyopathy
HOCM: Hypertrophic obstructive cardiomyopathy
HNCM: Hypertrophic non-obstructive cardiomyopathy
ACMG: American College of Medical Genetics and Genomics
LVOT: Left ventricular outflow tract
LVEF: Left ventricular ejection fraction
CMR: Cardiac magnetic resonance
NSVT: Non-sustained ventricular tachycardia
TTE: Transthoracic echocardiography
LVEDP: Left ventricular end-diastolic pressure
PRS: Polygenic risk scoring
eQTL: Expression quantitative trait locus
iPSC: Induced pluripotent stem cell
TTNtv: TTN truncation variants
GWAS: Genome-wide association studies
