# Correction: The MYH7 c.2770G > A (p.Glu924Lys) mutation exhibits phenotypic heterogeneity in hypertrophic cardiomyopathy (HCM) and restrictive cardiomyopathy (RCM): a case report

**DOI:** 10.1186/s12872-026-05603-4

**Published:** 2026-02-18

**Authors:** Yuanyuan Han, Haiyan Wang, Hongsheng Zhang, Manman Wang, Lijun Gan, Fanhua Meng

**Affiliations:** 1 Department of Cardiology, Affiliated Hospital of Jining Medical University, Jining, Shandong China; 2 Shandong Provincial Key Medical and Health Discipline of Cardiology Affiliated Hospital, Jining Medical University, Jining, Shandong China; 3 Key Laboratory of Cell and Biomedical Technology of Shandong Province, Jining, Shandong China

**Correction: BMC Cardiovascular Disorders 25, 514 (2025)**

**https://doi.org/10.1186/s12872-025-04943-x**

In this article [1], the author’s name Haiyan Wang was incorrectly written as Haiyang Wang.

The original article has been corrected.
