# Supplementary material for: The MYH7 c.2770G > A (p.Glu924Lys) mutation exhibits phenotypic heterogeneity in hypertrophic cardiomyopathy (HCM) and restrictive cardiomyopathy (RCM): a case report
Source: BMC Cardiovasc Disord. 2025 Jul 16;25:514. doi: 10.1186/s12872-025-04943-x (PMC12265364; doi:10.1186/s12872-025-04943-x)
Supplement: Supplementary file 1 — Supplementary Material 1 [file 12872_2025_4943_MOESM1_ESM.pdf]

The *MYH7* c.2770G>A (p.Glu924Lys) mutation exhibits phenotypic heterogeneity in hypertrophic cardiomyopathy (HCM) and restrictive cardiomyopathy (RCM): a case report

Yuanyuan Han[1,2,3#], Haiyang Wang[1,2#], Hongsheng Zhang[1,2,3], Manman Wang[1,2,3], Lijun Gan[1,2,3*], Fanhua Meng[1,2,3*]

[1]Department of Cardiology, Affiliated Hospital of Jining Medical University, Shandong, China

[2]Shandong Provincial Key Medical and Health Discipline of Cardiology Affiliated Hospital of Jining Medical University, Shandong, China,

[3]Key laboratory of cell and biomedical Technology of Shandong Province.

# These authors contributed equally to this work.

*Correspondence: Fanhua Meng (mengfanhuaxj@163.com)

Exome-wide analysis of mutations in the DES, FLNC, and CRYAB in HCM and RCM families.

Gene	II-2	II-4	II-6	III-3	III-4	III-5
DES	None	None	None	None	None	None
FLNC	None	None	None	None	None	None
CRYAB	None	None	None	None	None	None
LMNA	None	None	None	None	None	None
BAG3	None	None	None	None	None	None
TTN	None	None	None	None	None	None
MYL2	None	None	None	None	None	None
ACTC1	None	None	None	None	None	None
TNNT2	None	None	None	None	None	None
TNNI3	None	None	None	None	None	None
TPM1	None	None	None	None	None	None
MYL3	None	None	None	None	None	None
MYL2	None	None	None	None	None	None
MYPN	None	None	None	None	None	None
TTN	None	None	None	None	None	None
MYBPC3	None	None	None	None	None	None
TNNC1	None	None	None	None	None	None
TMEM87B	None	None	None	None	None	None
ACTN2	None	None	None	None	None	None
DCBLD2	None	None	None	None	None	None
